# Supplementary material for: Therapeutic Potential of Magnetic Nanoparticle-Based Human Adipose-Derived Stem Cells in a Mouse Model of Parkinson’s Disease
Source: Int J Mol Sci. 2021 Jan 11;22(2):654. doi: 10.3390/ijms22020654 (PMC7827941; doi:10.3390/ijms22020654)
Supplement: Supplementary file 1 [file ijms-22-00654-s001.pdf]

Supplementary Figures

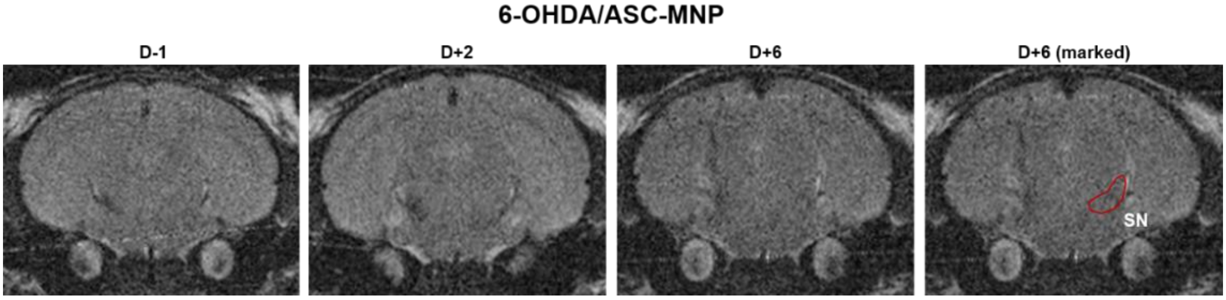

Supplementary Figure S1. Representative MR imaging of 6-OHDA PD mice injected with magnetic nanoparticles-labeled hASCs (6-OHDA/ASC-MNP).

MR images were acquired at optimized parameters on a 9.4T Bruker animal imager before injection (D-1), 2 and 6 days after injection (D+2 and D+6). Substantia nigra was indicated the area outlined in red on the D+6 image. MR imaging parameters were follows; Pulse sequence = Multiple Gradient Echo 2D, Echo train = 10, Selected image's Echo train = 2, Echo spacing = 3 ms, Effective TE (Echo time) = 6 ms, TR (Repetition time) = 2000 ms, FA (Flip Angle) = 50 degree, Matrix size = 256 x 256, Slice thickness = 0.7 mm, FOV (Field of view) = 15.36 x 17.92 mm^2, Pixel bandwidth = 520.83 pixel/Hz.

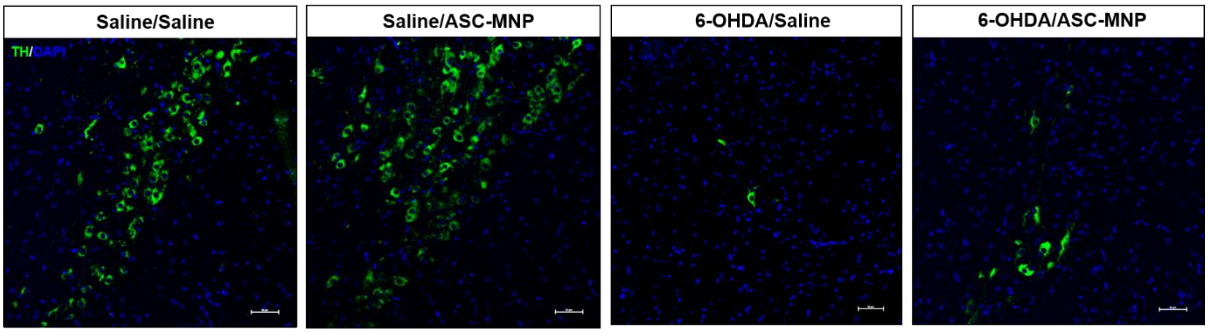

Supplementary Figure S2. Loss of dopaminergic neurons was decreased by the transplantation of hASC.

The brain slices were immunofluorescence stained with an anti-TH antibody (sc-14007, Santa Cruz). TH-positive dopaminergic neurons were visualized by Alexa Fluor® 488 donkey anti-rabbit IgG (H+L) antibody (A21206, Invitrogen). Dopaminergic neuronal cell death in the SN was sharply increased following the injection of 6-OHDA, and the dopaminergic neurons recovered with the hASC transplantation. Scale bar 50μm. Experimental groups: Sham/Saline; saline-injected sham mouse, Sham/ASC-MNP; hASCs-injected sham mouse, 6-OHDA/Saline; saline-injected 6-ODHA induced PD mouse, 6-OHDA/ASC-MNP; hASCs-injected 6-ODHA induced PD mouse.
